# Limited Specificity in the Injury and Infection Priming against Bacteria in *Aedes aegypti* Mosquitoes

**DOI:** 10.3389/fmicb.2016.00975

**Published:** 2016-06-22

**Authors:** Valeria Vargas, Miguel Moreno-García, Erika Duarte-Elguea, Humberto Lanz-Mendoza

**Affiliations:** ^1^Centro de Investigaciones Sobre Enfermedades Infecciosas, Instituto Nacional de Salud PúblicaCuernavaca, Mexico; ^2^Posgrado de Ciencias Biológicas, Universidad Nacional Autónoma de MéxicoMexico City, Mexico; ^3^Department of Microbiology, Immunology and Pathology, Colorado State UniversityFort Collins, CO, USA

**Keywords:** injury and infection priming, insect, antimicrobial peptides, susceptible-primed-infected (SPI), epidemiological model

## Abstract

Injury and infection priming has been observed in several insect groups, reported as host immune protection against contact with a pathogen caused by a previous infection with the same. However, the specific response against a pathogen has not been demonstrated in all insect species. Investigating the specific priming response in insects is important because their immune strategies probably reflect particular selective pressures exerted by different pathogens. Here, we determined whether previous infection of *Aedes aegypti* would enhance survival and/or lead to greater and specific AMP expression after a second exposure to the same or a distinct bacterium. Mosquitoes previously immunized with a low dose of *Escherichia coli*, but not *Staphylococcus aureus*, showed increased survival. Although the host protection herein demonstrated was not specific, each bacterium elicited differential AMP expression. These results can be explained by the susceptible-primed-infected (SPI) epidemiological model, which poses that in the evolution of memory-like responses (priming), a pivotal role is played by pathogen virulence, associated host damage, and the host capacity of pathogen recognition.

## Introduction

Injury and infection priming is a memory-like response that is reportedly elicited in several (but not all) insect groups by bacteria, fungi, viruses, parasites and even inert molecules (LPS, heat-killed bacteria) (Moret and Siva-Jothy, 2003; Sadd et al., 2005; Moret, 2006; Sadd and Schmid-Hempel, 2007; Roth et al., 2009; Hernández-Martínez et al., 2010; Tidbury et al., 2011; Tate and Rudolf, 2012; Christofi and Apidianakis, 2013). Despite the evidence of a widespread phenomenon, the mechanism(s) and molecule(s) associated with the induction of priming are as yet unclear (but see Contreras-Garduño et al., 2015; Ramirez et al., 2015).

There are studies showing that after previous exposure to a pathogen, there was specific protection against a subsequent lethal challenge. For instance, after previous contact of *Drosophila melanogaster* with different bacteria, individuals were able to increase its survival rate and specific phagocytic activity against the corresponding pathogen (Pham et al., 2007; Christofi and Apidianakis, 2013). While different strains of *Pseudomona aeruginosa* elicit defense-specific genes (Apidianakis et al., 2005). *Anopheles gambiae* showed dissimilar responses to *Plasmodium berghei* and *P. falciparum* (Rodrigues et al., 2010). However, these studies do not necessarily reveal the specific recognition of a pathogen, but highlights a specificity response degree against different bacteria during priming.

The specificity of the response against infectious agents can promote an expedited immune activation leading to elimination or control of the invader. The energetic cost of a specific response could be lower than that of a generic immune response or one that is continuously activated (Pham and Schneider, 2008). Consequently, the resources spared by the use of a specific immune response can be used for the expression of other traits linked with survival and reproduction.

There has been criticism of the reports posing that previous contact with an infectious agent can afford an insect with improved immunity against a second exposure to the same pathogen. The specificity of the insect memory-like immune response is rejected by some researchers (see Hauton and Smith, 2007) because of the lack of antibodies as well as clonal expansion and differentiation. Nonetheless, the immune system of insects comprises a number of humoral (soluble and membrane-associated) recognition receptors PRRs (Hoffmann and Reichhart, 2002; Strand, 2008). Moreover, the DSCAM is a PRR that can take thousands of different forms through alternative splicing (Watson et al., 2005; Armitage et al., 2014). In *A. gambiae*, Dscam (AgDscam) is capable of producing pathogen specific splice form repertoires upon bacteria (Gram+ and Gram-) and *Plasmodium* infection (Dong et al., 2006). Dscam plays a role mainly as an opsonization molecule with an increased affinity to the infectious organisms (Dong et al., 2006; Smith et al., 2011).

Recognition leads to the activation of three signaling pathways: Toll, IMD and the JAK/STAT (Hoffmann and Reichhart, 2002). In *Drosophila*, these pathways can induce a differential expression of AMPs following a bacterial challenge (Lemaitre et al., 1997; Apidianakis et al., 2005). The differential recognition of pathogens and production of effectors indicates a certain degree of specificity of the immune system.

Puzzlingly, not all insects seem to show this differential activation and regulation of immune pathways. For example, in *Aedes aegypti* cellular and melanization responses are independent of the bacterial Gram type (see Hillyer et al., 2004). Even more, some AMPs depend on two pathways (Levashina et al., 1998). At first glance, it could seem unlikely that selective AMP production provides an explanation for the subsequent specific protection in some insects. After all, when dipterans are re-infected, their immunity is mediated by granulocytes (Pham et al., 2007; Rodrigues et al., 2010). This through prohemocyte differentiation that increased the granulocyte population and trigger changes in the morphology and binding properties of these cells (Rodrigues et al., 2010), thus conferring enhanced immunity to following parasite infections.

However, there are signaling pathways required for hemocytes to differentiate in response to one or more differentiation factors (Rodrigues et al., 2010; Ramirez et al., 2014, 2015). Immune pathways of hemocytes are activated after pathogen recognition or humoral stimulation, which leads to the production and release of signaling and/or effector molecules, including AMPs (Bartholomay et al., 2004a). Hemocytes can also induce AMP expression in other tissues, such as fat body (a tissue with great AMP production) (Imler and Bulet, 2005). Consequently, it is plausible that increased cellular activity upon reinfection could have an effect on AMP production. In this sense, Contreras-Garduño et al. (2015) compared two groups of *A. albimanus* in contact with *P. berghei*. One had had prior contact with this pathogen and the other had not. There was greater AMP transcription in the previously infected mosquitoes. Therefore, it is probable that injury and infection priming entails specific cellular and humoral products, possibly interconnected with each other.

Previous studies have reported injury and infection priming in *A. aegypti* mosquito (Moreira et al., 2009; Bian et al., 2010; Moreno-García et al., 2015), revealing the role of immune pathways and the participation of AMPs in memory-like responses, meaning the ability to enhance or intensify the effectiveness of the immune response after a previous contact with an elicitor of the immune response. However, priming was analyzed by using consecutive challenges with the same pathogen, but without directly testing the specificity of the response. It is important to determine the specificity of the immune response in insects, because the selective pressures exerted by different pathogens could be reflected in their immune strategies.

**FIGURE 1:**
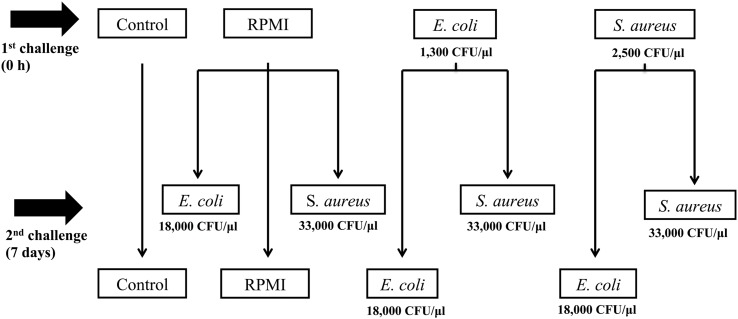
**Experimental design of *Aedes aegypti* immune priming challenge (first injection) and lethal challenge (second injection), using homologous and heterologous challenges with *Escherichia coli*, *Staphylococcus aureus*, and RPMI medium only**.

For this reason, the aim of the present study was to determine whether previous exposure to bacteria enhanced the survival of *A. aegypti* and/or led to greater AMP expression after a second exposure to the same or a distinct bacteria. For infections, *Escherichia coli* (Gram-) and *Staphylococcus aureus* (Gram+) were used as pathogen models. We documented AMP transcription at three different designated time points after a non-lethal dose (priming induction) and at another three time points following a second exposure 7 days post-priming (**Figure 1**). With this design, we tried to avoid overlap of the second challenge with the response generated by the priming dose.

The results demonstrate an increased survival of the mosquitoes previously immunized with a low dose of *E. coli*, but not with *S. aureus*. Although this protection was not specific, differential AMP expression proved to be elicited by each bacterium. We discuss these results in light of the SPI epidemiological model developed by Tidbury et al. (2012) and Best et al. (2013). In this model, pathogen virulence, associated host damage, and the host capacity of pathogen recognition have an essential role in the evolution of memory-like responses (priming).

## Materials and Methods

Female adult mosquitoes 3–5 days old were used for the experiments. They were reared under insectary conditions (12:12 h light/dark cycle at 25–28°C) at the Instituto National de Salud Pública (INSP), Mexico. The microbial strains employed for infection were the Gram+ bacterium *S. aureus* (the 1MR strain, which is methicillin and oxacillin-resistant, kindly donated by Dr. María Elena Velázquez, INSP) and the Gram- bacterium *E. coli* (the 01268 strain, which is ampicillin resistant, kindly donated by Dr. Jesús Silva, INSP). To reach the exponential growth phase, the two bacteria were incubated in LB-broth at 37°C (200 RPMI), the first for 4 h and the second for 3.15 h (for both bacteria final OD_600_ ≈ 0.350).

### Priming and Lethal Doses

The priming doses (LD_0_) were 2500 CFU/μl for *S. aureus* and 1300 CFU/μl for *E. coli*. For the lethal challenge (LD_30_), we used a higher dose of *E. coli* (18 × 10^3^ CFU/μl) and *S. aureus* (33 × 10^3^ CFU/μl). These doses were previously determined by injecting separate groups of mosquitoes with serial dilutions of each bacterium. We used RPMI (GIBCO-Na_2_CO_3_, 300 mg/L L-glutamine) as a vehicle for the bacterial injections. This medium was used as a vehicle because mosquito mortality attributable to injection is reduced with this cell culture medium when compared to Schneider and Grace’s insect medium (*Log-rank x*^2^= 131.006, *P* < 0.001), while no significant difference between PBS, Saline solution (0.9% NaCl) and MEM medium (*Log-rank x*^2^= 1.075–9.18, *P* > 0.05) (Supplementary Figure S1) was observed. RPMI has been used in other studies (see Herrera-Ortíz et al., 2004; Hernández-Martínez et al., 2013a,b; Moreno-García et al., 2015).

### Injections

Mosquitoes were cold (4°C) anesthetized and injected using a pulled glass needle attached to a Drummond Captrol III microinjector. The injection was applied in the abdomen close to the junction of the ventral and dorsal cuticles, volume injected about ≈ 0.1 μl per mosquito. After injections, mosquitoes were transferred to the insectary and maintained on *ad libitum* sucrose solution.

### Experimental Design

We first injected two groups of mosquitoes (*n* = 200) with a sublethal dose (LD_0_), using either *S. aureus* or *E. coli* (**Figure 1**) to trigger injury and infection priming. A third group (the positive control, *n* = 300) was injected with RPMI only (**Figure 1**). The negative control group (*n* = 100) was only cold anesthetized for 10 min.

We challenged the mosquitoes with the LD_30_ dose 7 days after the primary exposure (**Figure 1**). For this second exposure, half of the *E. coli* primed mosquitoes (*n* = 100) were injected with *E. coli* (the *E. coli–E. coli* group) and the other half (*n* = 100) with *S. aureus* (the *E. coli–S. aureus* group) in order to explore the specificity of the response. Likewise, half of the *S. aureus* primed group (*n* = 100) was given a second injection with *S. aureus* (the *S. aureus–S. aureus* group) and the other half (*n* = 100) with *E. coli* (the *S. aureus–E. coli* group) in order to explore the specificity of the response. The RPMI injected mosquitoes were divided into three groups for the second injection: the first was administered *E. coli* (the RPMI-*E. coli* group, *n* = 100), the second *S. aureus* (the RPMI-*S. aureus* group, *n* = 100), and the third RPMI only (the RPMI–RPMI group, *n* = 100; the positive control) (**Figure 1**). The negative control group (C) was only cold anesthetized again for 10 min.

### Survival of Adults after the Lethal Challenge

After the lethal challenge (second injection), survival was recorded for 29 days. A *Log-rank x*^2^ test was used to detect differences in survival curves between primed (with the same or a different bacterium), unprimed and control groups. The analysis was undertaken using JMP 7.0 (SAS Institute, 2007). Three different biological repetitions were developed.

### Antimicrobial Peptide Transcript Dynamics (qRT-PCR Analyses)

Before the priming-induction injection and at 10 h, 24 h, and 7 days post-priming (first injection), ten mosquitoes from each group were collected and stored at -70°C to await processing. Likewise, at 10 h, 24 h, and 7 days post-lethal challenge (second injection), ten mosquitoes from each group were collected and stored at -70°C until processed.

Total RNA was extracted from each of the groups using 500 μl Trizol reagent (*Invitrogen*) and RNA concentration was measured using Nanodrop. We used 500 ng/μl of the total RNA for cDNA synthesis using the RevertAid Premium Reverse Transcriptase (*Thermo Scientific*). The resultant cDNA was quantified and well-adjusted a 500 ng/μl, and 1μl was used for real-time quantitative PCR reactions. The qPCR reaction was performed using gene-specific primers for cecropin (*CEC* Id: AAEL015515-RA, 160 pb; forward 5′ TCA CAA AGT TAT TTC TCC TGA TCG 3′; reverse 5′ GCT TTA GCC CCA GCT ACA AC 3′), attacin (*ATA* Id: AAEL003389-RA, 231 pb; forward 5′ TTG GCA GGC ACG GAA TGT CTT G 3′; reverse 5′ TGT TGT CGG GAC CGG GAA GTG 3′), defensin (*DEF* Id: AAEL003832-RA, 200 pb; forward 5′ TTG TTT GCT TCG TTG CTC TTT 3′; reverse 5′ ATC TCC TAC ACC GAA CCC ACT 3′) *ribosomal protein S7* (internal control, Id: AAEL009496-RA, 190 pb; forward 5′ GGG ACA AAT CGG CCA GGC TAT C 3′, reverse 5′ TCG TGG ACG CTT CTG CTT GTT G 3′), and Maxima SYBR Green/ROX qPCR Master Mix (*Thermo Scientific*) on a StepOne Plus Real-Time PCR system (*Applied Biosystem*). These sequences were used previously in a priming study with *A. aegypti* (Moreno-García et al., 2015).

Relative quantification of mRNA levels was done by the 2*^-ΔΔC^_T_* method, and primer efficiencies were calculated by measuring how the standard *ΔC_T_* varied with template serial dilutions (PCR efficiency is about 95–99% for each AMP). For all trials, the ribosomal protein gene S7 was used as the reference. The levels of *CEC, DEF*, and *ATA* were normalized with respect to the S7 transcript of the same sample. Melting curve analyses confirmed that only cDNA, and not genomic DNA, was amplified. The relative expressions of AMP’s were represented in ratios of 0 to 1, because the differences between transcript expressions of AMP were extremely disproportional. Therefore, we homogenize these differences of copy number in ratios for the three AMP genes. Three independent trials were conducted, each analyzed in duplicate.

Differences between groups were analyzed with a one-way ANOVA. Where significant ANOVA differences were found, an LSD-Fisher *post hoc* was implemented to identify the nature of these differences. Values are presented as the mean ± SE. Analyses were carried out using Statistica 7.0 (StatSoft, Inc.).

## Results

### Survival

**FIGURE 2:**
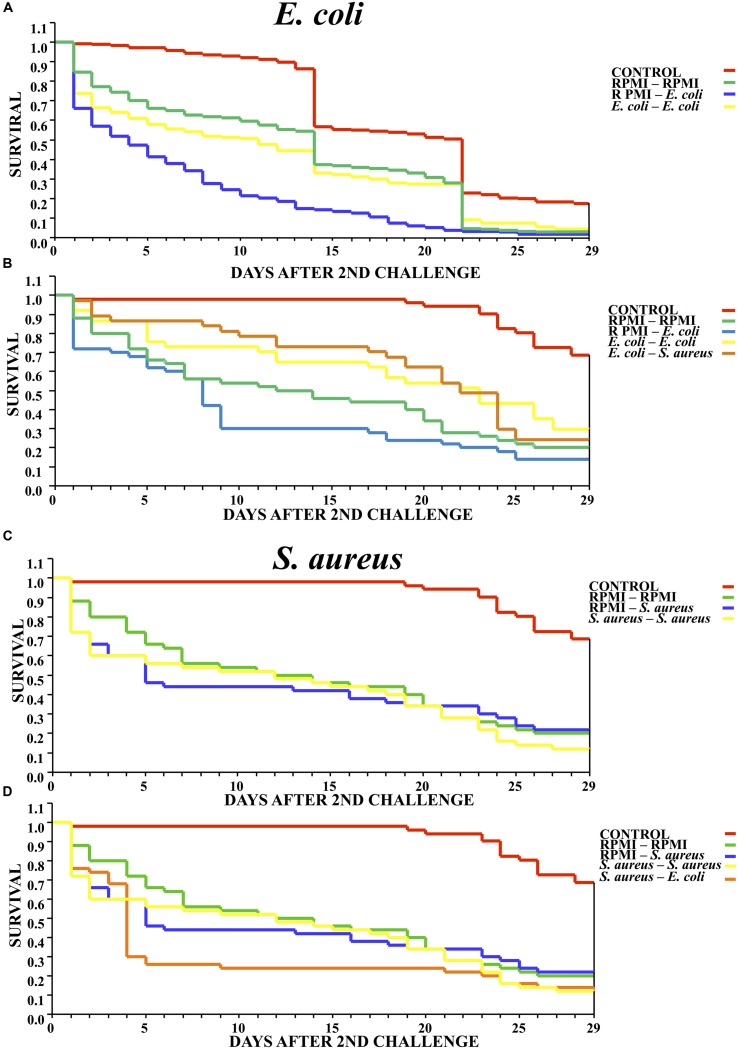
**Survival curves for mosquitoes after the second challenge, showing the results when using homologous and heterologous challenges with *E. coli*, *S. aureus* and RPMI medium only. (A)** Second injection with *E. coli* or RPMI medium only, after first injection with *E. coli* or RPMI medium only. **(B)** Second injection with *E. coli*, *S. aureus*, or RPMI medium only, after first injection with *E. coli* or RPMI medium only. **(C)** Second injection with *S. aureus* or RPMI medium only, after first injection with *S. aureus* or RPMI medium only. **(D)** Second injection with *S. aureus*, *E. coli*, or RPMI medium only, after first injection with S. *aureus* or RPMI medium only. Control were mosquitoes that were not treated (cold-only). Data are expressed as the mean ± SE from 3 independent experiments.

The survival analysis revealed that exposure to a low dose of *E. coli* delayed significantly mortality of mosquitos against the lethal challenge of the same bacterium. That is, there was a higher survival rate for the *E. coli–E. coli* than RPMI-*E. coli* group (*Log-rank x*^2^ = 32.143, *P* < 0.00001) (**Figure 2A**). The results are from three different biological repetitions. Moreover, priming with *E. coli* also provided protection against a lethal challenge with *S. aureus* (**Figure 2B**).

However, previous exposure to *S. aureus* did not elicit priming against the lethal challenge with the same bacterium, evidenced by the fact that the survival of the *S. aureus–S. aureus* mosquitoes did not differ from that of the RPMI-*S. aureus* group (*Log-rank x*^2^= 1.014, *P* = 0.602) (**Figure 2C**). Furthermore, previous exposure to *S. aureus* did not elicit a protective response against the lethal challenge with *E. coli* (*Log-rank x*^2^= 3.718, *P* = 0.293) (**Figure 2D**). Regarding the lethal challenge, *E. coli* showed a greater negative impact on the survival of mosquitoes than *S. aureus*, suggesting that impact of the former probably was continuous through time.

### Dynamics of Antimicrobial Peptide Transcripts (Response Induced by *E. coli* Priming)

**FIGURE 3:**
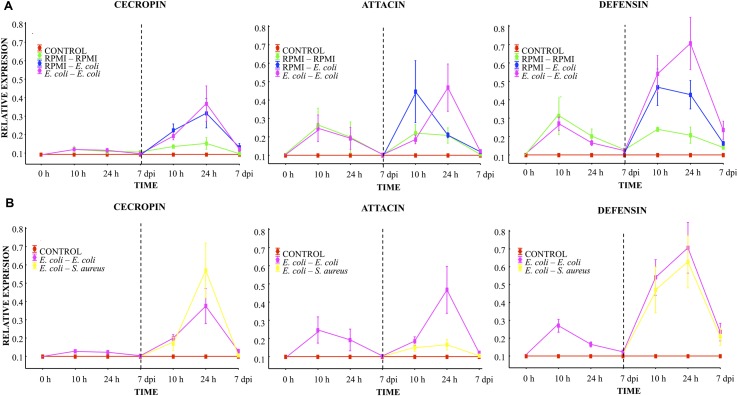
**Analysis of AMP expression in *A. aegypti* mosquitoes demonstrated an immune response with homologous and heterologous challenges using *E. coli*, *S. aureus*, and RPMI medium only. (A)** Second injection with *E. coli* or RPMI medium only, after first injection with *E. coli* or RPMI medium only. **(B)** Second injection with *E. coli* or *S. aureus*, after first injection with *E. coli*. Control were mosquitoes that were not treated (cold-only). Data are expressed as the mean ± SE from 3 independent experiments.

Before the first injection, no differences were observed in the relative mRNA levels of transcripts for any of the three peptides (cecropin, attacin or defensin, **Figure 3**). Ten hours after the first injection using RPMI or *E. coli*, a slight cecropin relative expression was observed in both cases. Attacin and defensin expression was higher after the first injection using RPMI or *E. coli*, although there were no differences between these two injected groups (RPMI vs. *E. coli*, **Figure 3A**). At 24 h post-injection, a decreased expression of the transcripts was observed for both groups, and at the 7th day post-injection values were close to 0.1.

Compared to the value found after the first injection, 10 h after the lethal challenge (second injection) with *E. coli* (in the *E. coli–E. coli* and RPMI-*E. coli* groups), there was an increased expression of cecropin and defensin (one and fourfold, respectively), with no differences between these two groups (**Figure 3A**). Attacin also showed an increased expression, which was higher for the RPMI-*E. coli* than the *E. coli–E. coli* group (LSD *Post hoc*, *P* = 0.002; **Figure 3A**; Supplementary Table S1). For the RPMI–RPMI group, the AMP expression was lower than that found in the other injected groups (**Figure 3A**). The increased AMP expression after the second injection can be attributed to the higher *E. coli* dose used for the lethal challenge than for priming. At 24 h post-lethal challenge (second injection), we observed a priming effect with regard to attacin and defensin, evidenced by the higher level of these peptides in the *E. coli–E. coli* than RPMI-*E. coli* group (LSD *post hoc*, *P* = 0.002 and *P* = 0.004, respectively; **Figure 3A**). Cecropin showed an increased expression in both these groups, but there was no difference between the *E. coli–E. coli* and RPMI-*E. coli* groups. For the RPMI–RPMI group, the AMP expression remained at a low level compared to the other injected groups. At the 7th day post-lethal challenge, the AMP expression in all groups declined to around 0.2 or 0.1, with no differences detected between groups.

At 10 h, 24 h, and 7 days post-lethal challenge, there were no significant differences in cecropin and defensin expression between the *E. coli–E. coli* and *E. coli–S. aureus* groups (**Figure 3B**). However, at 24 h post-lethal challenge, a lower expression of attacin was observed in the *E. coli–S. aureus* than *E. coli–E. coli* group (see **Figure 3B**; Supplementary Table S1). These results suggest a dissimilar AMP expression induced by each bacterium. Defensin and cecropin expression could be correlated with the observed enhanced survival found in both of these groups, while attacin expression only correlated with the survival of the *E. coli–E. coli* group.

### Dynamics of the Antimicrobial Peptide Transcripts (Response Induced by *S. aureus* Priming)

**FIGURE 4:**
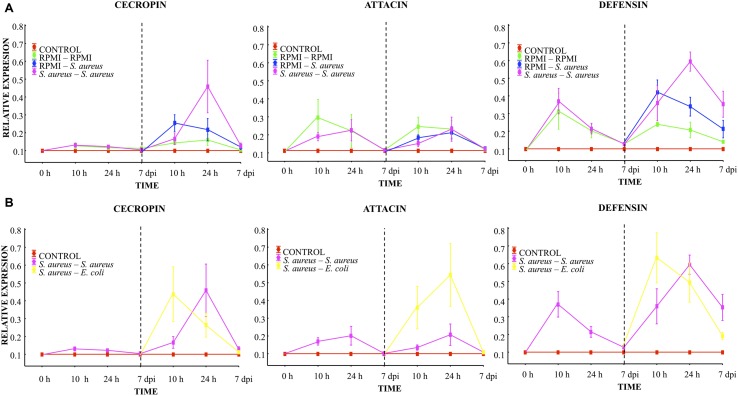
**Analysis of AMP expression in *A. aegypti* mosquitoes demonstrated an immune response with homologous and heterologous challenges using *E. coli*, *S. aureus* and RPMI medium only. (A)** Second injection with *S. aureus* or RPMI medium only, after first injection with *S. aureus* or RPMI medium only. **(B)** Second injection with *S. aureus* or *E. coli*, after first injection with *S. aureus*. Control were mosquitoes that were not treated (cold-only). Data are expressed as the mean ± SE from 3 independent experiments.

At 10 h, 24 h, and 7 days post-injection with the low dose of *S. aureus* (first injection), low AMP expression was observed. Attacin and defensin expression was higher than that of cecropin. However, no differences were found between the RPMI and *S. aureus* groups (**Figure 4A**). A decreased expression of the transcripts was observed in all groups at 24 h post-injection, reaching values close to 0.1 by the 7th day.

Compared to the expression after the first injection, 10 h after the lethal challenge a significant increase in defensin and a slight increment of cecropin were observed in both the *S. aureus–S. aureus* and RPMI-*S. aureus* groups, with no differences between groups (**Figure 4A**). We observed a priming effect at 24 h post-lethal challenge, evidenced by the higher levels of cecropin and defensin found in the *S. aureus–S. aureus* than RPMI-*S. aureus* group (LSD *post hoc*, *P* = 0.001 and *P* = 0.008, respectively; **Figure 4A**, Supplementary Table S1). No increased expression of attacin was detected at any time point for the RPMI-*S. aureus*, *S. aureus–S. aureus*, or *E. coli–S. aureus* group.

At 10 h post-lethal challenge, there was significantly greater cecropin and defensin expression for the *S. aureus–E. coli* than *S. aureus–S. aureus* group (**Figure 4B**; Supplementary Table S1). Likewise, 24 h after the lethal challenge the attacin expression was greater for the *S. aureus–E. coli* than *S. aureus–S. aureus* group (**Figure 4B**; Supplementary Table S1).

Injection damage (represented by the RPMI–RPMI group, Supplementary Table S2) triggered AMP expression. This expression showed the same trend after the first and second injection (**Figure 4A**). However, levels of expression after the second injection were below those of the first injection for all groups. This result and the low attacin expression in the *E. coli–S. aureus*, *S. aureus–S. aureus*, and RPMI-*S. aureus* groups suggest that *S. aureus* was unable to induce attacin expression.

## Discussion

The present study demonstrates that in females of *A. aegypti*, priming with a low dose of *E. coli* elicited a protective response against a subsequent lethal challenge with the same bacteria or with *S. aureus.* This protective response correlated with the expression of attacin and defensin.

Contrarily, exposure to a low dose of *S. aureus* did not improve the survival of mosquitoes after a lethal challenge with *S. aureus* or *E. coli*. However, there was an increased expression of defensin and cecropin in the group administered a low dose of *S. aureus* followed by a lethal challenge with the same bacterium. Additionally, there was an increased expression not only of defensin and cecropin but also of attacin in heterologous-challenged group (*S. aureus*–*E coli*), indicating that the two bacteria elicited a differential AMP expression. These results suggest a quasi-specificity (*sensu* Rowley and Powell, 2007) in AMP expression induced by the lethal challenge.

Although it has been demonstrated that some invertebrates express injury and infection priming (for one or more pathogens), and in some cases specificity of the immune response, evidence in other insect groups reveals that a memory-like response apparently does not exist (e.g., Pham et al., 2007; González-Tokman et al., 2010; Reber and Chapuisat, 2012; Dubuffet et al., 2015; Wu et al., 2015). Our results show that *S. aureus* induced priming in terms of the expression level of AMPs, but not in regard to survival. To our knowledge, this is the first report demonstrating this effect.

The non-ubiquitous expression of priming (specific or otherwise) in insects has been explained by the concept of the inducible costs of priming (Moret and Schmid-Hempel, 2000; González-Tokman et al., 2010). Likewise, the short life span of some insect groups limits the chance of a secondary encounter with the same pathogen (Little and Kraaijeveld, 2004). The SPI model proposes that pathogens producing damage select for greater priming to reduce the negative effects of the infection. But this also depends on the level of protection afforded against future exposure (Tidbury et al., 2012; Best et al., 2013).

In *Galleria mellonella*, (Mowlds et al., 2010) the authors demonstrated the existence of a threshold below which the immune response is aimed at solely containing the infection. Above this threshold, the immune response not only contains the infection but also protects the host from subsequent contact with the same pathogen. Hence, the host is capable of optimally tuning its response by sensing the infection levels. Our results show that after the lethal challenge, *E. coli* had greater impact than *S. aureus* (observed by the higher mortality of the RPMI-*E. coli* and *S. aureus–E. coli* groups), implying a greater chance of damage by this gram- bacteria. Contrarily, the *S. aureus–S. aureus*, RPMI-*S. aureus*, and RPMI–RPMI groups showed similar mortality curves (**Figure 2C**). In accordance with the SPI injury and infection priming theory, *E. coli* induced memory-like responses to prevent or diminish damage, leading to greater survival of mosquitoes after injection with a lethal dose.

Compared to *E. coli*, *S. aureus* apparently produced lower damage levels to the mosquitoes. Despite the increased cecropin and defensin levels in the *S. aureus–S. aureus* group after the second injection, and the effective activity of these AMPs against Gram-positive bacteria (Lowenberger et al., 1995, 1999; Bartholomay et al., 2004b; Bulet and Stöcklin, 2005), their expression was not sufficient to enhance the survival of primed mosquitoes. Since *S. aureus* had a low effect on the integrity of vital host processes, if mosquitoes were infected with a higher dose they would likely not sense any notable increase in damage, meaning that the organism should not make any increased investment in immune response without a survival compensation. The activation of the immune transcriptional pathways may be influenced by several factors, including damage, recognition of the microorganism responsible for producing the damage, and a recovery threshold (van Baalen, 1998; Moreno-García et al., 2014a). After the lethal challenge, the increased expression of defensin activated by *S. aureus* was probably related to the degree of damage to the mosquito, but not to a real compensation in terms of survival. Our results also showed that injury itself (RPMI–RPMI group) could have an impact on the survival, but not in AMPs expression. We hypothesize that mosquito cannot control damage without pathogen sensing. The immune response is turned off when pathogen and damage reach a threshold level where negative effects are “insignificant” and when no more danger molecules (self or non-self) are produced (Chambers and Schneider, 2012). Probably, the damage, without the bacteria, produces an impairment of the regulation of the immune response (ROS, PO but not AMP as figures showed) decreasing mosquitoes survival. Endogenous damage-associated molecular patterns (DAMPs) or danger signals associated with tissue damage can induce innate trained immunity through epigenetic regulation of transcriptional programs (Netea et al., 2011). According to the danger model, damage to tissues induce the release of DAMPs, allowing the proper type and level intensity of the immune response against a certain pathogen (Crisşan et al., 2016).

This trained immunity induced by previously encountered pathogens may also help to respond rapidly and appropriately to the next challenge. However, it seems that in *A. aegypti* the concurrence of damage and the pathogen leaded to an optimal immune response. Several DAMPs have been found in insects (Moreno-García et al., 2014b). It will be very interesting to determine the DAMPs particularly involved in the *A. aegypti*’s priming against bacteria.

Pathogen presence over several generations could be also required to generate a proper recognition and therefore an investment in priming (Best et al., 2013). In the wild, the microbiota of *A. aegypti* is mostly constituted by Gram-negative bacteria (Zouache et al., 2011; Ramirez et al., 2012). Therefore, it is possible that the mosquito is prone to a priming response when interacting with *E. coli* or other Gram-negative bacteria, while investment in priming and enhanced immunity could be quite costly in relation to the interaction between the mosquito and an uncommon Gram positive bacterium (such as *S. aureus*). The non-expression of attacin in the interaction between the mosquito and *S. aureus* could be related to the specificity of immunity, and particularly to the cost of production of attacin in response or to an infrequent gram+ bacterium (*S. aureus*). However, this explanation needs further research.

In summary, we have provided evidence for a quasi-specific injury and infection priming in *A. aegypti* at the AMP transcriptional level. However, molecular priming in this study did not always correlate with increased mosquito survival. It is likely that investment in the immune response depends on several factors, including pathogen virulence, level of infection, host damage, pathogen presence over several generations, and frequency of contact with the pathogen in the microenvironment of an insect. The mechanisms of immune response in insects include reactions and molecules that are interconnected. Therefore, we cannot exclude the possibility that the memory-like response also depended on cellular activity and/or other humoral molecules not measured herein. The present results suggest that not all the immune effectors may undergo a simultaneous increase in response to pathogens. Priming against bacteria seems to rely on the molecules released after damage (DAMPs) caused by infection, the level of protection afforded against future infections, and probably the prevalence of a pathogen across generations.

## Author Contributions

Conceived and designed the experiments: MM-G, VV, HL-M. Performed the experiments: MM-G, VV, ED-E. Analyzed the data: MM-G, VV. Contributed reagents/materials/analysis tools: HL-M. Wrote the paper: MM-G, VV, HL-M.

## Conflict of Interest Statement

The authors declare that the research was conducted in the absence of any commercial or financial relationships that could be construed as a potential conflict of interest.

## Abbreviations

AMP: antimicrobial peptide
CFU: colony forms units
DSCAM: down syndrome cell adhesion molecule
IMD: immune deficiency
JAK/STAT: janus kinase/signal transducers and activators of transcription
LD: lethal doses
PRR: pattern recognition receptor
RPMI: Roswell Park Memorial Institute
SPI: susceptible-primed-infected

## References

[B1] Apidianakis Y. Mindrinos M. N. Xiao W. Lau G. W. Baldini R. L. Davis R. W. (2005). Profiling early infection responses: *Pseudomonas aeruginosa* eludes host defenses by suppressing antimicrobial peptide gene expression. *Proc. Natl. Acad. Sci. U.S.A.* 102 2573–2578. 10.1073/pnas.0409588102 15695583 PMC549001

[B2] Armitage S. A. O. Peuß R. Kurtz J. (2014). Dscam and pancrustacean immune memory - a review of the evidence. *Dev. Comp. Immunol.* 48 315–323. 10.1016/j.dci.2014.03.004 24657209

[B3] Bartholomay L. C. Cho W. L. Rocheleau T. A. Boyle J. P. Beck E. T. Fuchs J. F. (2004a). Description of the transcriptomes of immune response-activated hemocytes from the mosquito vectors *Aedes aegypti* and *Armigeres subalbatus*. *Infect. Immun.* 72 4114–4126. 10.1128/IAI.72.7.4114-4126.2004 15213157 PMC427405

[B4] Bartholomay L. C. Fuchs J. F. Cheng L. L. Beck E. T. Vizioli J. Lowenberger C. (2004b). Reassessing the role of defensin in the innate immune response of the mosquito, *Aedes aegypti*. *Insect Mol. Biol.* 13 125–132. 10.1111/j.0962-1075.2004.00467.x 15056359

[B5] Best A. Tidbury H. White A. Boots M. (2013). The evolutionary dynamics of within-generation immune priming in invertebrate hosts. *J. R. Soc. Interface* 10:20120887 10.1098/rsif.2012.0887 PMC3565738 23269850

[B6] Bian G. Xu Y. Lu P. Xie Y. Xi Z. (2010). The endosymbiotic bacterium *Wolbachia* induces resistance to dengue virus in *Aedes aegypti*. *PLoS Pathog.* 6:e1000833 10.1371/journal.ppat.1000833 PMC2848556 20368968

[B7] Bulet P. Stöcklin R. (2005). Insect antimicrobial peptides: structures, properties and gene regulation. *Protein Pept. Lett.* 12 3–11. 10.2174/0929866053406011 15638797

[B8] Chambers M. C. Schneider D. S. (2012). Pioneering immunology: insect style. *Curr. Opin. Immunol.* 24 10–14. 10.1016/j.coi.2011.11.003 22188798

[B9] Christofi T. Apidianakis Y. (2013). Drosophila immune priming against *Pseudomonas aeruginosa* is short-lasting and depends on cellular and humoral immunity. *F1000Res.* 2:6 10.12688/f1000research.2-76.v1 PMC3752738 24358857

[B10] Contreras-Garduño J. Rodríguez M. C. Martínez S. H. Barnetche J. M. Alvarado A. Izquierdo J. (2015). *Plasmodium berghei* induced priming in *Anopheles albimanus* independently of bacterial co-infection. *Dev. Comp. Immunol.* 52 172–181. 10.1016/j.dci.2015.05.004 26004500

[B11] Crisşan T. Netea M. G. Joosten L. A. B. (2016). Innate immune memory: implications for host responses to damage-associated molecular patterns. *Eur. J. Immunol.* 4 817–828. 10.1002/eji.201545497 26970440

[B12] Dong Y. Taylor H. E. Dimopoulos G. (2006). AgDscam, a hypervariable immunoglobulin domain-containing receptor of the *Anopheles gambiae* innate immune system. *PLoS Biol.* 4:e229 10.1371/journal.pbio.0040229 PMC1479700 16774454

[B13] Dubuffet A. Zanchi C. Boutet G. Moreau J. Teixeira M. Moret Y. (2015). Trans-generational immune priming protects the eggs only against gram-positive bacteria in the mealworm beetle. *PLoS Pathog.* 11:e1005178 10.1371/journal.ppat.1005178 PMC4592268 26430786

[B14] González-Tokman D. M. González-Santoyo I. Lanz-Mendoza H. Córdoba Aguilar A. (2010). Territorial damselflies do not show immunological priming in the wild. *Physiol. Entomol.* 35 364–372. 10.1111/j.1365-3032.2010.00752.x

[B15] Hauton C. Smith V. J. (2007). Adaptive immunity in invertebrates: a straw house without a mechanistic foundation. *Bioessays* 29 1138–1146. 10.1002/bies.20650 17935208

[B16] Hernández-Martínez P. Naseri B. Navarro-Cerrillo G. Escriche B. Ferré J. Herrero S. (2010). Increase in midgut microbiota load induces an apparent immune priming and increases tolerance to *Bacillus thuringiensis*. *Environ. Microbiol.* 12 2730–2737. 10.1111/j.1462-2920.2010.02241.x 20482744

[B17] Hernández-Martínez S. Barradas-Bautista D. Rodríguez M. H. (2013a). Diferential dna synthesis in *Anopheles albimanus* tissues induced by immune challenge with different microorganisms. *Arch. Insect Biochem. Physiol.* 84 1–14. 10.1002/arch.21108 23797988

[B18] Hernández-Martínez S. Lanz-Mendoza H. Martínez-Barnetche J. Rodríguez M. H. (2013b). Antimicrobial properties of *Anopheles albimanus* pericardial cells. *Cell Tissue Res.* 351 127–137. 10.1007/s00441-012-1505-6 23229355 PMC3536983

[B19] Herrera-Ortíz A. Lanz-Mendoza H. Martínez-Barnetche J. Hernández-Martínez S. Villarreal-Treviño H. Aguilar-Marcelino L. (2004). *Plasmodium berghei* ookinetes induce nitric oxide production in Anopheles pseudopunctipennis midguts cultured in vitro. *Insect Biochem. Mol. Biol.* 34 893–901. 10.1016/j.ibmb.2004.05.007 15350609

[B20] Hillyer J. F. Schmidt S. L. Christensen B. M. (2004). The antibacterial innate immune response by the mosquito *Aedes aegypti* is mediated by hemocytes and independent of Gram type and pathogenicity. *Microbes Infect.* 6 448–459. 10.1016/j.micinf.2004.01.005 15109959

[B21] Hoffmann J. A. Reichhart J. M. (2002). Drosophila innate immunity: an evolutionary perspective. *Nat. Immunol.* 3 121–126. 10.1038/ni0202-121 11812988

[B22] Imler J. L. Bulet P. (2005). “Antimicrobial peptides in *Drosophila*: structures, activities and gene regulation,” in *Mechanisms of Epithelial Defense*, eds Kabelitz D. Schröder J. -M. (Basel: Karger), 1–21. 10.1159/000086648 15976485

[B23] Lemaitre B. Reichhart J. M. Hoffmann J. (1997). Drosophila host defense: differential induction of antimicrobial peptide genes after infection by various classes of microorganisms. *Proc. Natl. Acad. Sci. U.S.A.* 94 14614–14619. 10.1073/pnas.94.26.14614 9405661 PMC25070

[B24] Levashina E. Ohresser S. Lemaitre B. Imler J. L. (1998). Two distinct pathways can control expression of the gene encoding the *Drosophila antimicrobial* peptide metchnikowin. *J. Mol. Biol.* 278 515–527. 10.1006/jmbi.1998.1705 9600835

[B25] Little T. J. Kraaijeveld R. (2004). Ecological and evolutionary implications of immunological priming ininvertebrates. *Trends Ecol. Evol.* 19 58–60. 10.1016/j.tree.2003.11.011 16701227

[B26] Lowenberger C. Bulet P. Charlet M. Hetru C. Hodgeman B. Christensen B. M. (1995). Insect immunity: isolation of three novel inducible antibacterial defensins from the vector mosquito, *Aedes aegypti*. *Insect Biochem. Mol. Biol.* 25 867–873. 10.1016/0965-1748(95)00043-U 7633471

[B27] Lowenberger C. A. Kamal S. Chiles J. Paskewitz S. Bulet P. Hoffmann J. A. (1999). Mosquito-Plasmodium interactions in response to immune activation of the vector. *Exp. Parasitol.* 91 59–69. 10.1006/expr.1999.4350 9920043

[B28] Moreira L. A. Iturbe-Ormaetxe I. Jeffery J. A. Lu G. Pyke A. T. Hedges L. M. (2009). A *Wolbachia* symbiont in *Aedes aegypti* limits infection with Dengue, Chikungunya, and Plasmodium. *Cell* 139 1268–1278. 10.1016/j.cell.2009.11.042 20064373

[B29] Moreno-García M. Condé R. Bello-Bedoy R. Lanz-Mendoza H. (2014a). The damage threshold hypothesis and the immune strategies of insects. *Infect. Genet. Evol. J.* 24 25–33. 10.1016/j.meegid.2009.04.004 24614506

[B30] Moreno-García M. Récio-Totóro B. Claudio Piedras F. Lanz-Mendoza H. (2014b). Injury and immune response: applying the danger theory to mosquitoes. *Front. Plants Sci.* 5:451 10.3389/fpls.2014.00451 PMC4158974 25250040

[B31] Moreno-García M. Vargas V. Ramírez-Bello I. Hernández-Martínez G. Lanz-Mendoza H. (2015). Bacterial exposure at the larval stage induced sexual immune dimorphism and priming in adult *Aedes aegypti* Mosquitoes. *PLoS ONE* 10:e0133240 10.1371/journal.pone.0133240 PMC4504673 26181517

[B32] Moret Y. (2006). “Trans-generational immune priming”: specific enhancement of the antimicrobial immune response in the mealworm beetle, Tenebrio molitor. *Proc. Biol. Sci.* 273 1399–1405. 10.1098/rspb.2006.3465 16777729 PMC1560290

[B33] Moret Y. Schmid-Hempel P. (2000). Survival for immunity: the price of immune system activation for bumblebee workers. *Science* 290 1166–1168. 10.1126/science.290.5494.1166 11073456

[B34] Moret Y. Siva-Jothy M. T. (2003). Adaptive innate immunity? Responsive-mode prophylaxis in the mealworm beetle, Tenebrio molitor. *Proc. Biol. Sci.* 270 2475–2480. 10.1098/rspb.2003.2511 14667338 PMC1691523

[B35] Mowlds P. Coates C. Renwick J. Kavanagh K. (2010). Dose-dependent cellular and humoral responses in *Galleria mellonella* larvae following β-glucan inoculation. *Microbes Infect.* 12 146–153. 10.1016/j.micinf.2009.11.004 19925881

[B36] Netea M. G. Quintin J. van der Meer J. W. M. (2011). Trained immunity: a memory for innate host defense. *Cell Host Microbe* 9 335–361. 10.1016/j.chom.2011.04.006 21575907

[B37] Pham L. N. Dionne M. S. Shirasu-Hiza M. Schneider D. S. (2007). A specific primed immune response in *Drosophila* is dependent on phagocytes. *PLoS Pathog.* 3:e26 10.1371/journal.ppat.0030026 PMC1817657 17352533

[B38] Pham L. N. Schneider D. (2008). “Evidence for specificity and memory in the insect innate immune response,” in *Insect Immunology*, ed. Beckage N. (San Diego, CA: Academic Press), 97–127. 10.1016/B978-012373976-6.50007-0

[B39] Ramirez J. L. de Almeida Oliveira G. Calvo E. Dalli J. Colas R. A. Serhan C. N. (2015). A mosquito lipoxin/lipocalin complex mediates innate immune priming in *Anopheles gambiae*. *Nat. Commun.* 6:7403 10.1038/ncomms8403 PMC4542143 26100162

[B40] Ramirez J. L. Garver L. S. Brayner F. A. Alves L. C. Rodrigues J. Molina-Cruz A. (2014). The role of hemocytes in *Anopheles gambiae* antiplasmodial immunity. *J. Innate Immun.* 6 119–128. 10.1159/000353765 23886925 PMC3901800

[B41] Ramirez J. L. Souza-Neto J. Cosme R. T. Rovira J. Ortiz A. Pascale J. M. (2012). Reciprocal tripartite interactions between the *Aedes aegypti* midgut microbiota, innate immune system and dengue virus influences vector competence. *PLoS Negl. Trop. Dis.* 6:e1561 10.1371/journal.pntd.0001561 PMC3295821 22413032

[B42] Reber A. Chapuisat M. (2012). No evidence for immune priming in ants exposed to a fungal pathogen. *PLoS ONE* 7:e35372 10.1371/journal.pone.0035372 PMC3327680 22523588

[B43] Rodrigues J. Brayner F. A. Alves L. C. Dixit R. Barillas-Mury C. (2010). Hemocyte differentiation mediates innate immune memory in *Anopheles gambiae* mosquitoes. *Science* 329 1353–1355. 10.1126/science.1190689 20829487 PMC3510677

[B44] Roth O. Sadd B. M. Schmid-Hempel P. Kurtz J. (2009). Strain-specific priming of resistance in the red flour beetle, Tribolium castaneum. *Proc. Biol. Sci.* 276 145–151. 10.1098/rspb.2008.1157 18796392 PMC2614262

[B45] Rowley A. F. Powell A. (2007). Invertebrate immune systems specific, quasi-specific, or nonspecific? *J. Immunol.* 179 7209–7214. 10.4049/jimmunol.179.11.7209 18025161

[B46] Sadd B. Schmid-Hempel P. (2007). Facultative but persistent trans- generational immunity via the mother’ s eggs in bumblebees. *Curr. Biol.* 17 1046–1047. 10.1016/j.cub.2007.11.007 18088585

[B47] Sadd B. M. Kleinlogel Y. Schmid-Hempel R. Schmid-Hempel P. (2005). Trans-generational immune priming in a social insect. *Biol. Lett.* 1 386–388. 10.1098/rsbl.2005.0369 17148213 PMC1626361

[B48] Smith P. H. Mwangi J. M. Afrane Y. A. Yan G. Obbard D. J. Ranford-Cartwright L. C. (2011). Alternative splicing of the *Anopheles gambiae* Dscam gene in diverse Plasmodium falciparum infections. *Malar. J.* 10:156 10.1186/1475-2875-10-156 PMC3118162 21651790

[B49] Strand M. R. (2008). “Insect hemocytes and their role in immunity,” in *Insect Immunology*, ed. Beckage N. E. (Cambridge, MA: Academic Press), 25–48.

[B50] Tate A. T. Rudolf V. H. W. (2012). Impact of life stage specific immune priming on invertebrate disease dynamics. *Oikos* 121 1083–1092. 10.1111/j.1600-0706.2011.19725.x

[B51] Tidbury H. J. Best A. Boots M. (2012). The epidemiological consequences of immune priming. *Proc. R. Soc. B Biol. Sci.* 279 4505–4512. 10.1098/rspb.2012.1841 PMC3479815 22977154

[B52] Tidbury H. J. Pedersen A. B. Boots M. (2011). Within and transgenerational immune priming in an insect to a DNA virus. *Proc. Biol. Sci.* 278 871–876. 10.1098/rspb.2010.1517 20861049 PMC3049047

[B53] van Baalen M. (1998). Coevolution of recovery ability and virulence. *Proc. Biol. Sci.* 265 317–325. 10.1098/rspb.1998.0298 9523434 PMC1688890

[B54] Watson F. L. Püttmann-Holgado R. Thomas F. Lamar D. L. Hughes M. Kondo M. (2005). Extensive diversity of Ig-superfamily proteins in the immune system of insects. *Science* 309 1874–1878. 10.1126/science.1116887 16109846

[B55] Wu G. Yi Y. Sun J. Li M. Qiu L. (2015). No evidence for priming response in Galleria mellonella larvae exposed to toxin protein PirA2B2 from *Photorhabdus luminescens* TT01: an association with the inhibition of the host cellular immunity. *Vaccine* 33 6307–6313. 10.1016/j.vaccine.2015.09.046 26432910

[B56] Zouache K. Raharimalala F. N. Raquin V. Tran-Van V. Raveloson L. H. R. Ravelonandro P. (2011). Bacterial diversity of field-caught mosquitoes, *Aedes albopictus* and *Aedes aegypti*, from different geographic regions of Madagascar. *FEMS Microbiol. Ecol.* 75 377–389. 10.1111/j.1574-6941.2010.01012.x 21175696

