# Supplementary material for: Limited Specificity in the Injury and Infection Priming against Bacteria in Aedes aegypti Mosquitoes
Source: Front Microbiol. 2016 Jun 22;7:975. doi: 10.3389/fmicb.2016.00975 (PMC4916184; doi:10.3389/fmicb.2016.00975)

Supplementary Material

*Limited specificity in the immune priming against bacteria in Aedes aegypti mosquitoes.*

Valeria Vargas ^1, 2^, Miguel Moreno-García^1, 3,^ *, Erika Duarte-Elguea^1^, Humberto Lanz-Mendoza ^1,^ *

*** Correspondence:** Corresponding Author: humberto@insp.mx; miguelmoga2000@yahoo.com.mx

# Supplementary Tables

**Supplementary Table 1.** Different combinations of groups in the expression of antimicrobial peptides at 10 h, 24 h and 7 days after the second challenge with *E. coli* and *S. aureus*.

| Defensin expression after the second challenge | | | | | | |  |
| --- | --- | --- | --- | --- | --- | --- | --- |
|  | ***E. coli-E. coli***  **10 h** | | ***E. coli-E. coli***  **24 h** | | ***E. coli-E. coli***  **7 days** | |  |
| RPMI-*E. coli* 10 h | NS | | - | | - | |  |
| RPMI-*E. coli* 24 h | - | | higher in *E. coli-E. coli*  (P = 0.004) | | - | |  |
| RPMI-*E. coli* 7 days | - | | - | | NS | |  |
|  |  | |  | |  | |  |
| *E. coli-S. aureus* 10 h | NS | | - | | - | |  |
| *E. coli-S. aureus* 24 h | - | | NS | | - | |  |
| *E. coli-S. aureus* 7 days | - | | - | | NS | |  |
|  |  | |  | |  | |  |
| RPMI-RPMI 10 h | higher in *E. coli-E. coli*  (P = 0.002) | | - | | - | |  |
| RPMI-RPMI 24 h | - | | higher in *E. coli-E. coli*  (P = 0.0000) | | - | |  |
| RPMI-RPMI 7 days | - | | - | | NS | |  |
|  |  | |  | |  | |  |
|  | ***S. aureus-S. aureus***  **10 h** | | ***S. aureus-S. aureus***  **24 h** | | ***S. aureus-S. aureus***  ***7 days*** | |  |
| RPMI-*S. aureu*s 10 h | NS | | - | | - | |  |
| RPMI-*S. aureu*s 24 h | - | | higher in *S. aureus-S. aureus* (P = 0.008) | | - | |  |
| RPMI-*S. aureus* 7 days | - | | - | | NS | |  |
|  |  | |  | |  | |  |
| *S. aureus-E. coli* 10 h | higher in *S. aureus-E. coli* (P = 0.004) | | - | | - | |  |
| *S. aureus-E. coli* 24 h | - | | NS | | - | |  |
| *S. aureus-E. coli* 7 days | - | | - | | NS | |  |
|  |  | |  | |  | |  |
| RPMI-RPMI 10h | NS | | - | | - | |  |
| RPMI-RPMI 24h | - | | higher in *S. aureus-S. aureus* (P = 0.0000) | | - | |  |
| RPMI-RPMI 7days | - | | - | | higher in *S. aureus-S. aureus* (P = 0.02) | |  |
|  |  | |  | |  | |  |
|  | ***E. coli-E. coli***  **10 h** | | ***E. coli-E. coli***  **24 h** | | ***E. coli-E. coli***  **7 days** | |  |
| *S. aureus-S. aureus* 10 h | | NS | | - | | - | |
| *S. aureus-S. aureus* 24 h | | - | | NS | | - | |
| *S. aureus-S. aureus* 7 days | | - | | - | | NS | |

NS. No significant between group.

| Attacin expression after the second challenge | | | |
| --- | --- | --- | --- |
|  | ***E. coli-E. coli***  **10 h** | ***E. coli-E. coli***  **24 h** | ***E. coli-E. coli***  **7 days** |
| RPMI-*E. coli* 10 h | higher in RPMI-*E. coli*  (P = 0.002) | - | - |
| RPMI-*E. coli* 24 h | - | higher in *E. coli-E.coli*  (P = 0.002) | NS |
| RPMI-*E. coli* 7 days | - | - | NS |
|  |  |  |  |
| *E. coli-S. aureus* 10 h | NS | - | - |
| *E. coli-S. aureus* 24 h | - | higher in *E. coli-E.coli*  (P = 0.0003) | - |
| *E. coli-S. aureus* 7 days | - | - | NS |
|  |  |  |  |
| RPMI-RPMI 10 h | NS | - | - |
| RPMI-RPMI 24 h | - | higher in *E. coli-E.coli*  (P = 0.002) | - |
| RPMI-RPMI 7 days | - | - | NS |
|  |  |  |  |
|  | ***S. aureus-S. aureus***  **10 h** | ***S. aureus-S. aureus***  **24 h** | ***S. aureus-S. aureus***  ***7 days*** |
| RPMI-*S. aureu*s 10 h | NS | - | - |
| RPMI-*S. aureu*s 24 h | - | NS | - |
| RPMI-*S. aureus* 7 days | - | - | NS |
|  |  |  |  |
| *S. aureus-E. coli* 10 h | higher in *S. aureus-E. coli*  (P = 0.007) | - | - |
| *S. aureus-E. coli* 24 h | -- | higher in *S. aureus-E. coli*  (P = 0.0000) |  |
| *S. aureus-E. coli* 7 days | - | - | NS |
|  |  |  |  |
| RPMI-RPMI 10h | NS | - | - |
| RPMI-RPMI 24h | - | NS | - |
| RPMI-RPMI 7days | - | - | NS |
|  |  |  |  |
|  | ***E. coli-E. coli***  **10 h** | ***E. coli-E. coli***  **24 h** | ***E. coli-E. coli***  **7 days** |
| *S. aureus-S. aureus* 10 h | NS | - | - |
| *S. aureus-S. aureus* 24 h | - | higher in *E. coli-E.coli*  (P = 0.002) | - |
| *S. aureus-S. aureus* 7 days | - | - | NS |

NS. No significant between group.

| Cecropin expression after the second challenge | | | | |
| --- | --- | --- | --- | --- |
|  | ***E. coli-E. coli***  **10 h** | ***E. coli-E. coli***  **24 h** | ***E. coli-E. coli***  **7 days** |  |
| RPMI-*E. coli* 10 h | NS | - | - |  |
| RPMI-*E. coli* 24 h | - | NS | - |  |
| RPMI-*E. coli* 7 days | - | - | NS |  |
|  |  |  |  |  |
| *E. coli-S. aureus* 10 h | NS | - | - |  |
| *E. coli-S. aureus* 24 h | - | higher in *E. coli-S. aureus*  (P = 0.008) | - |  |
| *E. coli-S. aureus* 7 days | - | - | NS |  |
|  |  |  |  |  |
| RPMI-RPMI 10 h | NS | - | - |  |
| RPMI-RPMI 24 h | - | higher in *E. coli-E.coli*  (P = 0.003) | - |  |
| RPMI-RPMI 7 days | - | - | NS |  |
|  |  |  |  |  |
|  | ***S. aureus-S. aureus***  **10 h** | ***S. aureus-S. aureus***  **24 h** | ***S. aureus-S. aureus***  ***7 days*** |  |
| RPMI-*S. aureu*s 10 h | NS | - | - |  |
| RPMI-*S. aureu*s 24 h | - | higher in *S. aureus-S. aureus* (P = 0.001) | - |  |
| RPMI-*S. aureus* 7 days | - | - | NS |  |
|  |  |  |  |  |
| *S. aureus-E. coli* 10 h | higher in *S. aureus-E. coli*  (P = 0.0003) | - | - |  |
| *S. aureus-E. coli* 24 h | - | higher in *S. aureus-S. aureus*  (P = 0.008) | - |  |
| *S. aureus-E. coli* 7 days | - | - | NS |  |
|  |  |  |  |  |
| RPMI-RPMI 10h | NS | - | - |  |
| RPMI-RPMI 24h | - | higher in *S. aureus-S. aureus* (P = 0.0000) | - |  |
| RPMI-RPMI 7days | - | - | NS |  |
|  |  |  |  |  |
|  | ***E. coli-E. coli***  **10 h** | ***E. coli-E. coli***  **24 h** | ***E. coli-E. coli***  **7 days** |  |
| *S. aureus-S. aureus* 10 h | NS | - | - |  |
| *S. aureus-S. aureus* 24 h | - | NS | - |  |
| *S. aureus-S. aureus* 7 days | - | - | NS |  |

NS. No significant between group.

**Supplementary Table 2.** Different combination of groups in survival curves after second challenge with simple and not infection with *E. coli.*

| Multiples comparison of survival curves | | | |
| --- | --- | --- | --- |
|  | ***Log-rank x^2^*** | ***P < value*** | ***Significant*** |
| CONTROL vs. RPMI-*E. coli* | 263.884 | 0.0000 | Yes |
| CONTROL vs. RPMI-RPMI | 65.188 | 0.0000 | Yes |
| CONTROL vs. *E. coli*-*E. coli* | 62.176 | 0.0000 | Yes |
| RPMI-RPMI vs. RPMI-*E. coli* | 62.017 | 0.0000 | Yes |
| RPMI-*E. coli* vs. *E. coli*-*E. coli* | 32.143 | 0.0000 | Yes |
| RPMI-RPMI vs. *E. coli*-*E. coli* | 0.236 | NS | No |

NS. No significant between group. All pairwise multiple comparison procedures by Bonferroni method.

**2 Supplementary Figures**

**Supplementary Figure 1.** Survival curves for mosquitoes after different solutions inoculation. Control were mosquitoes that were not treated (cold-only). Data are expressed as the mean ± standard error from 2 independent experiments.


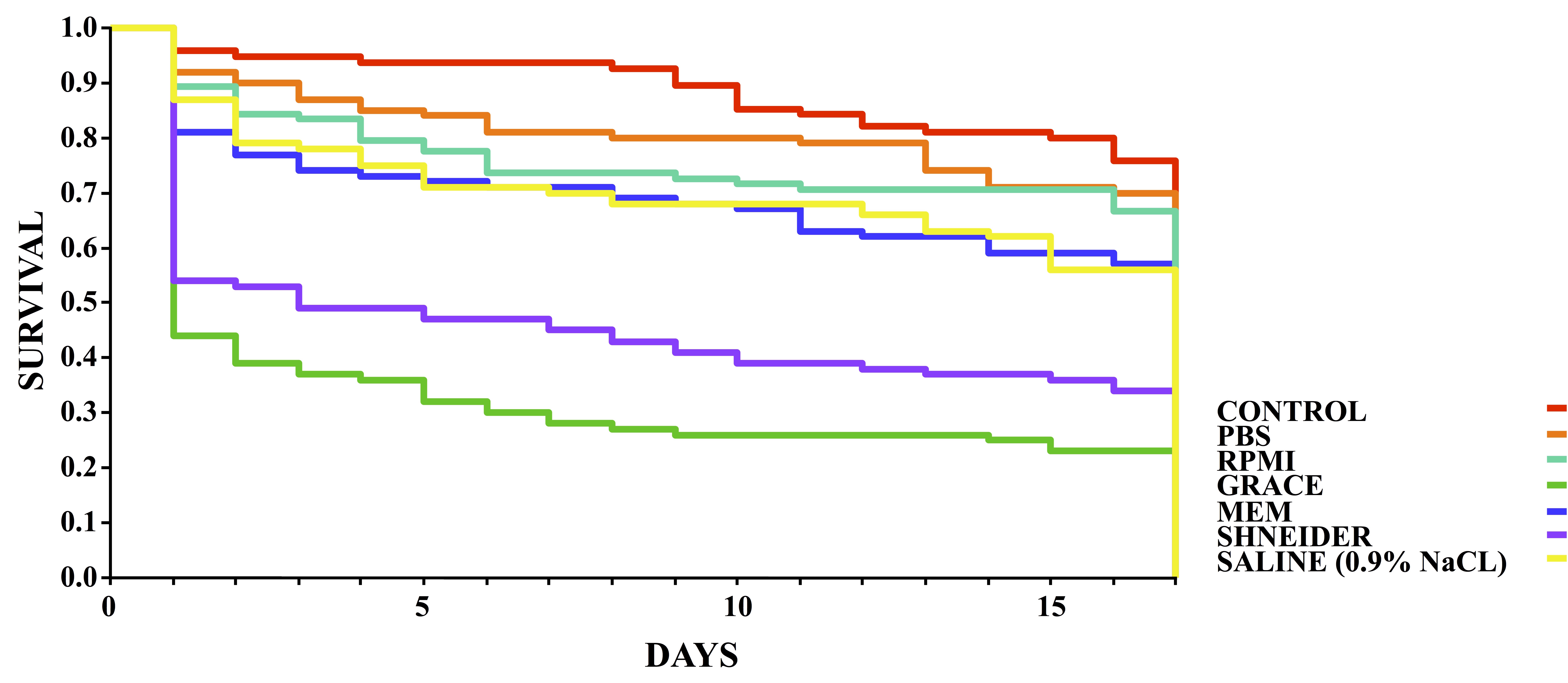

Supplement: Supplementary file 1 [file Data_Sheet_1.DOCX]
